# Role of nuclear factor of activated T cells 1 in the pathogenesis of osteoarthritis

**DOI:** 10.3892/etm.2013.1390

**Published:** 2013-11-07

**Authors:** RONGBIN SUN, BO ZHANG, LONG CHEN, JUNYING SUN

**Affiliations:** 1 Department of Orthopedics, The First Affiliated Hospital of Soochow University, Suzhou, Jiangsu 215006, P.R. China; 2 Department of Orthopedics, The Second People’s Hospital of Changzhou, Changzhou, Jiangsu 213003, P.R. China

**Keywords:** osteoarthritis, nuclear factor of activated T cells 1, pathogenesis

## Abstract

Osteoarthritis (OA) is the most common form of joint disease in middle-aged individuals and the elderly. Previous studies have shown that the overexpression of matrix-degrading proteinases and proinflammatory cytokines is associated with the degradation of osteoarthritic cartilage. However, the transcription factors involved remain unclear. The present study aimed to determine the expression levels of nuclear factor of activated T cells 1 (NFAT1), interleukin-1β (IL-1β) and tumor necrosis factor-α (TNF-α) in patients with OA, and to validate the role of NFAT1 in the pathogenesis of OA. The expression levels of NFAT1, IL-1β and TNF-α in chondrocytes in the cartilage of patients with OA and healthy individuals were evaluated using western blot analysis. A luciferase reporter assay was performed to determine the activity of NFAT1 in primary human chondrocytes that were transfected with pNFAT1-luc plasmid and stimulated by IL-1β. An enzyme-linked immunosorbent assay was performed to detect the levels of TNF-α, matrix metalloproteinase (MMP)-1, MMP-3 and MMP-9 in the supernatant of cultured chondrocytes in which the NFAT1 was silenced. The expression levels of NFAT1, IL-1β and TNF-α in the cartilage of patients with OA were higher than those of the controls. IL-1β induced the expression of NFAT1 in primary chondrocytes. The expression levels of TNF-α, MMP-1, -3 and -9 promoted by IL-1β were decreased in NFAT1-silenced chondrocytes. In conclusion, NFAT1 may be important in the pathogenesis of OA and calcineurin-NFAT inhibitors may be potential effective agents for the treatment of OA.

## Introduction

Osteoarthritis (OA), which involves the dysfunction of adult articular cartilage, is the most common form of joint disease with manifestations of damaged articular cartilage, chondro-osteophyte formation and thickening of subchondral bone, and may result in arthralgia, joint deformation and limited mobility in patients. OA is the second leading cause of long-term disability in adults (1).

Previous studies have shown that the synthetic activity of articular chondrocytes is dependent on insulin-like growth factor-1 (IGF-1), transforming growth factor-β (TGF-β) and bone morphogenetic protein (2–4), and controlled by interleukin-1β (IL-1β), tumor necrosis factor-α (TNF-α) and nitric oxide (NO) (5). OA often results from an imbalance in the catabolic and anabolic activity of cartilage and results in cartilage degradation. Numerous scientists have studied the imbalance of cartilage metabolism, but the signaling pathways involved remain unclear (5).

Nuclear factor of activated T cells 1 (NFAT1), also named NFATc2/NFATp, is a member of the NFAT family and was initially identified as a regulator of cytokine expression during the immune response (6). Early studies demonstrated that tumor-like proliferation appeared in the articular cartilage and peripheral joint tissues of adult mice deficient in NFAT1. However, gene mutation analysis identified that NFAT1 was not a tumor suppressor (6). A histopathological study found that mature NFAT1-deficient mice demonstrated manifestations of OA, such as articular chondrocytes decomposing to form clusters, bone bud formation and sub-chondral bone thickening (7). The manifestations of OA in mice are similar to those in humans (8,9). The primary aim of the present study was to clarify the role of NFAT1 in OA pathology.

## Subjects and methods

### Subjects

Twelve articular cartilage samples were collected from seven patients with OA and five additional patients who required joint-replacement surgery following a traffic accident. Cartilage tissues were collected from the First Affiliated Hospital of Soochow University (Suzhou, China) and preserved in liquid nitrogen within 6 h following the surgery to enable the analysis of NFAT1, IL-1β and TNF-α expression by western blotting. This study was conducted in accordance with the declaration of Helsinki and with approval from the Ethics Committee of the First Affiliated Hospital of Soochow University (Suzhou, China). Written informed consent was obtained from all participants.

### Isolation and culture of cartilage cells

Articular cartilage tissues from the healthy human knee were cut into 1–2 cm pieces with a scalpel, digested with trypsin (100 g/l) for 30 min followed by hyaluronidase (1 mg/ml; Sigma-Aldrich, Gillingham, UK) for 15 min and then incubated in collagen enzyme B (Roche Diagnostics Ltd., West Sussex, UK) for 12–15 h at 37ºC. The acquired cells were filtered through a 200 mesh copper screen. The cartilage cells were washed twice with phosphate-buffered saline (PBS) and cultured in Dulbecco’s modified Eagle’s medium (DMEM)/F12 (10% fetal bovine serum, 100 U/ml penicillin and 100 μg/ml streptomycin) to a concentration of 2×10^5^cells/ml. Chondrocytes were identified by morphological observation following the method of Li *et al*(10).

### Luciferase reporter assays

An equal number of chondrocytes (1×10^5^ per well) were seeded in each well of a 6-well plate. The cells were allowed to attach for 24 h. The cells were then transfected with a pNFAT1-luc plasmid (5 μg/ml, provided by Dr Weilin King of Shanghai Jiao Tong University, Xuhui, China) using Lipofectamine 2000 reagent (Invitrogen Life Technologies, Carlsbad, CA, USA) according to the manufacturer’s instructions. Cells transfected with an empty pGL3 vector and PBS were used as the control. Following transfection (24 h), the cells were incubated in 10 ng/ml IL-1β for 36 h. Then, cell extracts were analyzed for luciferase activity using the dual-luciferase reporter assay system (Promega Corp., Madison, WI, USA). The experiment was repeated three times and results were expressed as the mean ± standard error of the mean.

### RNAi-mediated gene silencing

An equal number of chondrocytes (1×10^5^ per well) were seeded into each well of a 6-well plate. The cells were allowed to attach for 24 h to 70–80% confluence. The cells were then transfected with siRNA targeting NFAT1 (5 μg/ml; 5′-CAGCGGAGTCCAAGGTTGTGTTCAT-3′) using Lipofectamine 2000 reagent. The control cells were transfected with scrambled siRNA in the presence of PBS. After 24 h, the cells were incubated at 37ºC with IL-1β (10 ng/ml) for 36 h. The supernatant was collected and TNF-α, MMP-1, MMP-3 and MMP-9 were detected by enzyme-linked immunosorbent assay (ELISA). The ELISA kits were obtained from R&D Systems Inc., Minneapolis, MN, USA (TNF-α), Amersham Pharma Biotech, Cambridge, England (MMP-1 and MMP-3) and R&D Systems Inc. (MMP-9). All of the experiments for each sample were performed in triplicate. Simultaneously, the total protein content was extracted using cell lysis buffer [0.3% NP-40, 1 mM EDTA, 50 mM Tris-Cl (pH 7.4), 2 mM EGTA, 1% Triton X-100, 150 mM NaCl, 25 mM NaF, 1 mM Na_3_VO_3_ and 10 μg/ml PMSF] and the expression of NFAT1 and β-actin were analyzed by western blotting according to the manufacturer’s instructions.

### Statistical analysis

Data were analyzed using SPSS software, version 11.0 (SPSS, Inc., Chicago, IL, USA) and are expressed as the mean ± standard error. Student’s t-test was performed for comparisons between two groups. The significant level was α=0.05 and P<0.05 was considered to indicate a statistically significant difference.

## Results

### Expression levels of NFAT1, IL-1β and TNF-α in OA

**Figure 1:**
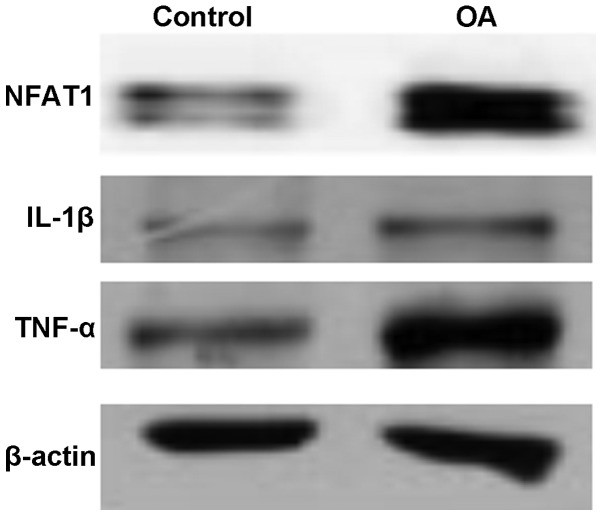
Expression levels of NFAT1, IL-1β and TNF-α in the articular cartilage of patients with OA and healthy controls. NFAT1, nuclear factor of activated T cells 1; IL-1β, interleukin-1β; TNF-α, tumor necrosis factor-α; OA, osteoarthritis.

The expression levels of NFAT1, IL-1β and TNF-α in the articular cartilage of patients with OA were higher than those in the healthy individuals. As shown in Fig. 1, the levels of phosphorylated and non-phosphorylated NFAT1 were observed to be higher in the patients with OA than in the control group, implying that OA may be associated with the transcription factor NFAT1. The expression levels of pro-inflammatory cytokines, IL-1β and TNF-α, were also increased in the patients with OA, and were positively correlated with NFAT1.

### Activation of NFAT1 is mediated by IL-1β

**Figure 2:**
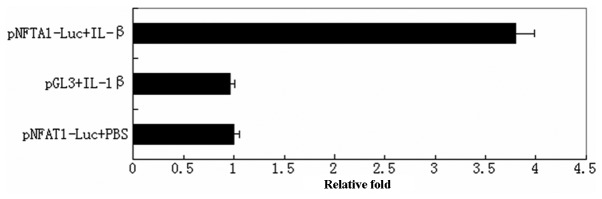
NFAT1 activity detected by a luciferase reporter assay. NFAT1, nuclear factor of activated T cells 1.

It is known that pro-inflammatory cytokines, such as IL-1β and TNF-α, play a role in the pathogenesis of osteoarthritis; therefore, the present study established a model of OA using normal human articular cartilage cells stimulated by recombinant human IL-1β. The model was used to evaluate the role of IL-1β in NFAT1 activation using the luciferase reporter assay method. As shown in Fig. 2, IL-1β significantly induced the activity of NFAT1 3-fold compared with that of the control group (P<0.05).

### Silencing of NFAT1 reduced the expression of TNF-α and MMPs

**Figure 3:**
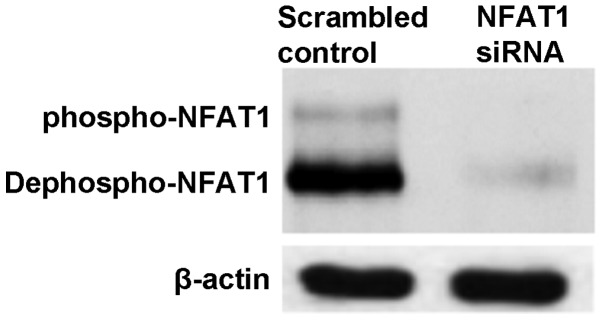
Silencing of NFAT1 in cultured chondrocyte cells. NFAT1, nuclear factor of activated T cells 1.

NFAT1 in cultured chondrocyte cells was silenced using NFAT1-siRNA (Fig. 3). Cells were then stimulated with IL-1β and the levels of TNF-α, MMP-1, -3 and -9 in the supernatant were detected by ELISA. As shown in Table I, silencing of NFAT1 weakened the IL-1β-induced stimulation of TNF-α, MMP-1, -3 and -9 secretion (P<0.05).

## Discussion

The NFAT family, which consists of five members, comprises the most important substrates of calcineurin (CaN). NFAT1 (NFATp), NFAT2 (NFATc) and NFAT4 (NFATx) predominantly exist in T cells, while NFAT3 exists in other tissues, such as the heart. NFAT5 is the original member of the NFAT family and is important for permeability responses (11–13). NFAT proteins consist of a trans-activation domain on the N-terminal, and the NFAT homology region (NHR), also known as the region of accommodation comprises nine conserved motifs, a highly conserved Rel region (RSD) and a C terminal. NFAT proteins differ from other phosphatase substrates in that they include ≥13 phosphorylation sites distributed in regions of accommodation and spanning a range of nearly 400 amino acids (12). The special structure of NFAT allows CaN to rapidly remove several phosphoric acids from the N terminal of NFATc protein, subsequently causing conformation alteration and exposure of the nuclear-localization sequence resulting in the rapid transposition of NFATc to the nucleus (10,14). The CaN-NFAT pathway may be blocked by inhibitors of CaN, such as cyclosporine (CsA) and FK506 (15,16). In the nucleus, NFATs participate in transcriptional regulation and cooperate with other transcription factors, such as AP-1 family members.

It is accepted that OA originates from the softening of articular cartilage due to the actions of catabolic enzymes, such as MMPs and dextranase. This results in slight inflammation and increased levels of TNF-α and IL-1β, which further stimulates the expression of catabolic enzymes and induces the transformation of subchondral bone and articular peripheral cartilage (5). However, the transcription factors involved in this transformation have not been identified. In the present study, the expression levels of NFAT1, IL-1β and TNF-α were evaluated in articular cartilage from patients with OA and healthy individuals using western blot analysis. The results demonstrated that the expression levels of NFAT1, IL-1β and TNF-α were significantly increased in the cartilage tissues of patients with OA (Fig. 1). The findings of this study are in accordance with the results of previous studies (16). Subsequently, a luciferase reporter assay demonstrated that treatment with IL-1β significantly induced the activity of NFAT1 in cultured human chondrocytes, indicating that the CaN-NFAT pathway may be activated by IL-1β (Fig. 3). The hypothesis is further validated by the silencing of NFAT1. In chondrocyte cells in which NFAT1 was silenced, the levels of TNF-α and MMPs induced by IL-1β decreased significantly compared with those of the normal chondrocyte cells, indicating that the CaN-NFAT pathway is closely associated with OA. However, it remains unclear whether additional pathways, such as nuclear factor-κ-light-chain-enhancer of activated B cells (NF-κB), are important in this process. Notably, there is a Rel domain that preserves a DNA-binding sequence with homology to NF-κB in the regulatory region of NFATs (17). Further studies are required to explore their roles of enhancing the proliferation and differentiation of cartilage in OA.

Although the contribution of the CaN-NFAT pathway to cartilage biology and OA remains unclear, a number of studies have shown that the CaN-NFAT pathway may be important for the proliferation and differentiation of cartilage and in reducing the activity of catabolic enzymes (18). The results of present study also support the hypothesis that inhibitors of CaN-NFAT may be effective in the treatment of OA.

## Figures and Tables

**Table I:** Silencing of NFAT1 in cultured chondrocyte cells decreased the levels of TNF-α and MMPs stimulated by IL-1β.

Group	TNF-α	MMP-1	MMP-3	MMP-9
Scrambled control	0.93±0.10	0.95±0.06	0.92±0.07	1.04±0.12
NFAT1-siRNA	0.63±0.05^a^	0.71±0.07^a^	0.66±0.03^a^	0.58±0.04^b^

a P<0.05,

b P<0.01 vs. the control.

NFAT1, nuclear factor of activated T cells 1; TNF-α, tumor necrosis factor-α; MMP, matrix metalloproteinase; IL-1β, interleukin-1β.
